# Psychological Interventions to Improve Upper Limb Motor Dysfunction Post-stroke: A Scoping Review

**DOI:** 10.7759/cureus.76784

**Published:** 2025-01-02

**Authors:** Yuji Iwamoto, Takeshi Imura, Tsubasa Mitsutake, Shingo Taki, Hungu Jung, Keiko Ogawa, Ryo Tanaka

**Affiliations:** 1 Department of Rehabilitation, Hiroshima Cosmopolitan University, Hiroshima, JPN; 2 Graduate School of Humanities and Social Sciences, Hiroshima University, Hiroshima, JPN; 3 Clinical Research Center, Saga University Hospital, Saga, JPN; 4 Department of Rehabilitation, Araki Neurosurgical Hospital, Hiroshima, JPN; 5 Department of Medicine for Integrated Approach to Social Inclusion, Hiroshima University, Hiroshima, JPN

**Keywords:** psychological interventions, randomized controlled trial (rct), scoping review, stroke, upper limb function

## Abstract

Cognitive strategies in post-stroke patients significantly influence upper limb motor function recovery. Integrating upper extremity and psychological interventions may enhance rehabilitation outcomes. This scoping review aimed to summarize studies evaluating the effectiveness of combining these approaches to improve upper extremity motor dysfunction in patients with post-stroke syndrome. Randomized controlled trials (RCTs) comparing combined upper extremity and psychological interventions versus upper extremity interventions alone were included. Studies published between November 25, 2024, and the study’s conclusion were retrieved from PubMed, Cochrane Central Register of Controlled Trials (CENTRAL), Physiotherapy Evidence Database (PEDro), and Cumulative Index to Nursing and Allied Health Literature. Only English-language studies were reviewed. Three RCTs met the inclusion criteria. Two studies utilized cognitive orientation to daily occupational performance (CO-OP), while one employed cognitive-oriented strategy training augmented rehabilitation (COSTAR). The CO-OP studies demonstrated that combined psychological and physical interventions significantly improved motor function compared to physical interventions alone. However, the COSTAR-based study reported greater efficacy for upper extremity interventions alone. This review highlighted the mixed efficacy of combined interventions. While CO-OP showed potential benefits, the COSTAR findings suggest variability in the effectiveness of different cognitive strategies. Both approaches prioritized activity and goal setting rather than directly targeting motor recovery. Although the findings are inconclusive, this is the first review to explore the role of combined psychological and upper extremity interventions for post-stroke motor dysfunction, providing a foundation for further research.

## Introduction and background

Improving upper extremity motor dysfunction is crucial in post-stroke rehabilitation. Severe sequelae in the upper extremity affect 43%-69% of patients with post-stroke motor dysfunction [1,2]. Post-stroke upper extremity motor dysfunction limits activities of daily living [3] and social reintegration [4] and causes an economic burden [5]. Furthermore, post-stroke upper extremity dysfunction is correlated with anxiety, low quality of life, and a higher incidence of disability [6,7]. Only 5%-20% of patients achieve complete improvement in upper extremity motor dysfunction six months post-stroke despite the importance of improving upper extremity motor dysfunction [8,9]. Therefore, clinicians are required to select and provide patients with appropriate interventions to improve upper limb motor dysfunction.

Various upper limb interventions have improved upper limb motor dysfunction in stroke patients. Upper extremity motor dysfunction improvement, which requires sufficient physical activity and high-quality upper limb interventions, involves the basal ganglia, cerebellum, and cerebral cortex [10]. Upper limb interventions induce structural plasticity changes in the nervous system, which develops compensatory neural networks and improves motor dysfunction [11]. A previous guideline has determined constraint-induced movement therapy, electrical stimulation, and mirror therapy with a high evidence level to improve upper extremity motor dysfunction [12]. Therefore, interventions with a high evidence level aimed at enhancing upper limb motor dysfunction have already been determined.

Stroke patients often experience not only motor dysfunction but also mental status changes, such as post-stroke depression [13] and cerebrovascular dementia [14]. These mental status changes influence participation in rehabilitation and the overall recovery process [15-17]. Accordingly, it is hoped that improvements in mental state will lead to improvements in motor function and ADL, as well as improvements in the effectiveness of rehabilitation. Therefore, interventions addressing upper limb motor dysfunction also need to consider psychological aspects. Psychological interventions that use cognitive strategies have been recommended for improving skill transfer and subsequent functioning and participation [18-20]. Previous research on cognitive strategies has described them as goal-oriented, consciously controllable processes that facilitate or support performance as subjects develop internal procedures for them to perform desired skills [21]. Interventions using cognitive strategies have helped improve motor dysfunction [22,23]. However, evidence for psychological interventions to enhance post-stroke upper extremity motor dysfunction is inconsistent in previous guidelines [12]. Consistent evidence on psychological interventions remains lacking despite the importance of psychological interventions in improving motor dysfunction.

Previous systematic reviews revealed the effects of psychological interventions on improving mental health in patients with post-stroke syndrome, but not on improving motor dysfunction [24]. However, the usefulness of combining upper extremity interventions with psychological interventions for post-stroke upper extremity motor dysfunction remains unclear. Furthermore, various psychological intervention methods, including emotional [13], cognitive [25], motivation [26,27], planning and executing [28] aspects, are available, and summarizing the interventions that are effective when combined with upper extremity interventions is important. This scoping review aimed to comprehensively investigate and summarize the usefulness of a combination of upper extremity and psychological interventions for upper extremity motor dysfunction in stroke patients. Our overarching research questions were “Is it useful to combine upper extremity interventions with psychological interventions for upper extremity motor dysfunction post-stroke?” and “What psychological interventions are useful?”

## Review

Methods 

Arksey and O’Malley’s scoping review framework [29] and the Preferred Reporting Items for Systematic Reviews and Meta-Analyses Scoping Review (PRISMA-ScR) reporting recommendations guided this scoping review methodology [30]. This scoping review was registered in the University Hospital Medical Information Network Clinical Trial Registry (ID: UMIN000045223).

Search Strategy and Selection Criteria

PubMed, Cochrane Central Register of Controlled Trials (CENTRAL), Physiotherapy Evidence Database (PEDro), and Cumulative Index to Nursing and Allied Health Literature electronic database searches were conducted to determine relevant studies and create a comprehensive list of references for the scoping review from the earliest available records to November 25, 2024. The search terms were combined with the “AND” operator. “Patient” was defined as a patient with post-stroke syndrome, “intervention” as psychotherapy and therapeutic exercise, “outcome” as upper extremity and motor function, and “research design” was limited to randomized controlled trials (RCTs). We combined synonyms and medical subject heading terms with the “OR” operator for each concept (Table 1).

**Table 1:** Search strategy

			Recent queries in PubMed			
			Search	Query	Items found	Time
RCT			#20	Search (#4 and #15 and #18) Filters: Randomized Controlled Tria	147	3:43:46
P and I and O			#19	Search (#4 and #15 and #18)	655	3:43:01
O	Upper limb motor function		#18	Search (#16 or #17) ((((((((((Upper Extremity[Title/Abstract]) OR (Extremities, Upper[Title/Abstract])) OR (Upper Extremities[Title/Abstract])) OR (Membrum superius[Title/Abstract])) OR (Upper Limb[Title/Abstract])) OR (Limb, Upper[Title/Abstract])) OR (Limbs, Upper[Title/Abstract])) OR (Upper Limbs[Title/Abstract])) OR (Extremity, Upper[Title/Abstract])) OR (Upper Extremity[MeSH Terms])) OR ((motor function[Title/Abstract]) OR (motor function[MeSH Terms]))	272,255	3:40:53
		Motor function	#17	(motor function[Title/Abstract]) OR (motor function[MeSH Terms])	41,525	3:40:17
		Upper extremity	#16	(((((((((Upper Extremity[Title/Abstract]) OR (Extremities, Upper[Title/Abstract])) OR (Upper Extremities[Title/Abstract])) OR (Membrum superius[Title/Abstract])) OR (Upper Limb[Title/Abstract])) OR (Limb, Upper[Title/Abstract])) OR (Limbs, Upper[Title/Abstract])) OR (Upper Limbs[Title/Abstract])) OR (Extremity, Upper[Title/Abstract])) OR (Upper Extremity[MeSH Terms])	235,363	3:39:45
I	Psychotherapy and therapeutic exercise		#15	Search (#10 and #14)	43,837	3:39:06
	Therapeutic exercise		#14	Search (#8 or #9 or#10) ((Rehabilitation[Title/Abstract]) OR (Habilitation[Title/Abstract])) OR (Rehabilitation[MeSH Terms])OR (((((Exercise[Title/Abstract]) OR (Physical Activity[Title/Abstract])) OR (Physical Exercise[Title/Abstract])) OR (Acute Exercise[Title/Abstract])) OR (Exercise Training[Title/Abstract])) OR (Exercise[MeSH Terms])OR(((Exercise therapy[Title/Abstract]) OR (Rehabilitation Exercise[Title/Abstract])) OR (Remedial Exercise[Title/Abstract])) OR (Exercise therapy[MeSH Terms])	1,015,637	3:37:46
		Exercise therapy	#13	(((Exercise therapy[Title/Abstract]) OR (Rehabilitation Exercise[Title/Abstract])) OR (Remedial Exercise[Title/Abstract])) OR (Exercise therapy[MeSH Terms])	71,872	3:37:12
		Exercise	#12	(((((Exercise[Title/Abstract]) OR (Physical Activity[Title/Abstract])) OR (Physical Exercise[Title/Abstract])) OR (Acute Exercise[Title/Abstract])) OR (Exercise Training[Title/Abstract])) OR (Exercise[MeSH Terms])	571,493	3:36:41
		Rehabilitation	#11	((Rehabilitation[Title/Abstract]) OR (Habilitation[Title/Abstract])) OR (Rehabilitation[MeSH Terms])	536,374	3:36:05
	Psychotherapy		#10	Search (#5 or #6 or #7 or #8) ((((((Psychotherapy[Title/Abstract]) OR (Psychotherapies[Title/Abstract])) OR (Psychotherapy[MeSH Terms])) OR (((((((((Psychological intervention[Title/Abstract]) OR (Intervention, Psychosocial[Title/Abstract])) OR (Interventions, Psychosocial[Title/Abstract])) OR (Psychosocial Interventions[Title/Abstract])) OR (Psychological Intervention[Title/Abstract])) OR (Intervention, Psychological[Title/Abstract])) OR (Interventions, Psychological[Title/Abstract])) OR (Psychological Interventions[Title/Abstract])) OR (Psychological intervention[MeSH Terms]))) OR (((((((((((((((((((((((((Cognitive behavioral therapy[Title/Abstract]) OR (Behavioral Therapies, Cognitive[Title/Abstract])) OR (Behavioral Therapy, Cognitive[Title/Abstract])) OR (Cognitive Behavioral Therapies[Title/Abstract])) OR (Therapies, Cognitive Behavioral[Title/Abstract])) OR (Therapy, Cognitive Behavioral[Title/Abstract])) OR (Therapy, Cognitive Behavior[Title/Abstract])) OR (Cognitive Behavior Therapy[Title/Abstract])) OR (Cognitive Therapy[Title/Abstract])) OR (Behavior Therapy, Cognitive[Title/Abstract])) OR (Behavior Therapies, Cognitive[Title/Abstract])) OR (Cognitive Behavior Therapies[Title/Abstract])) OR (Therapies, Cognitive Behavior[Title/Abstract])) OR (Cognitive Psychotherapy[Title/Abstract])) OR (Cognitive Psychotherapies[Title/Abstract])) OR (Psychotherapies, Cognitive[Title/Abstract])) OR (Psychotherapy, Cognitive[Title/Abstract])) OR (Therapy, Cognitive[Title/Abstract])) OR (Cognitive Therapies[Title/Abstract])) OR (Therapies, Cognitive[Title/Abstract])) OR (Cognition Therapy[Title/Abstract])) OR (Therapy, Cognition[Title/Abstract])) OR (Cognition Therapies[Title/Abstract])) OR (Therapies, Cognition[Title/Abstract])) OR (Cognitive behavioral therapy[MeSH Terms]))) OR ((((((((((((Behaviour therapy[Title/Abstract]) OR (Behavior Therapies[Title/Abstract])) OR (Therapy, Conditioning[Title/Abstract])) OR (Conditioning Therapy[Title/Abstract])) OR (Conditioning Therapies[Title/Abstract])) OR (Therapy, Behavior[Title/Abstract])) OR (Behavior Treatment[Title/Abstract])) OR (Treatment, Behavior[Title/Abstract])) OR (Behavior Modification[Title/Abstract])) OR (Behavior Modifications[Title/Abstract])) OR (Modification, Behavior[Title/Abstract])) OR (Behaviour therapy[MeSH Terms]))) OR (((((((((((((((((((((((((Cognitive therapy[Title/Abstract]) OR (Behavioral Therapies, Cognitive[Title/Abstract])) OR (Behavioral Therapy, Cognitive[Title/Abstract])) OR (Cognitive Behavioral Therapies[Title/Abstract])) OR (Therapies, Cognitive Behavioral[Title/Abstract])) OR (Therapy, Cognitive Behavioral[Title/Abstract])) OR (Therapy, Cognitive Behavior[Title/Abstract])) OR (Cognitive Behavior Therapy[Title/Abstract])) OR (Cognitive Therapy[Title/Abstract])) OR (Behavior Therapy, Cognitive[Title/Abstract])) OR (Behavior Therapies, Cognitive[Title/Abstract])) OR (Cognitive Behavior Therapies[Title/Abstract])) OR (Therapies, Cognitive Behavior[Title/Abstract])) OR (Cognitive Psychotherapy[Title/Abstract])) OR (Cognitive Psychotherapies[Title/Abstract])) OR (Psychotherapies, Cognitive[Title/Abstract])) OR (Psychotherapy, Cognitive[Title/Abstract])) OR (Therapy, Cognitive[Title/Abstract])) OR (Cognitive Therapies[Title/Abstract])) OR (Therapies, Cognitive[Title/Abstract])) OR (Cognition Therapy[Title/Abstract])) OR (Therapy, Cognition[Title/Abstract])) OR (Cognition Therapies[Title/Abstract])) OR (Therapies, Cognition[Title/Abstract])) OR (Cognitive therapy[MeSH Terms]))	303,217	3:23:45
		Cognitive therapy	#9	((((((((((((((((((((((((Cognitive therapy[Title/Abstract]) OR (Behavioral Therapies, Cognitive[Title/Abstract])) OR (Behavioral Therapy, Cognitive[Title/Abstract])) OR (Cognitive Behavioral Therapies[Title/Abstract])) OR (Therapies, Cognitive Behavioral[Title/Abstract])) OR (Therapy, Cognitive Behavioral[Title/Abstract])) OR (Therapy, Cognitive Behavior[Title/Abstract])) OR (Cognitive Behavior Therapy[Title/Abstract])) OR (Cognitive Therapy[Title/Abstract])) OR (Behavior Therapy, Cognitive[Title/Abstract])) OR (Behavior Therapies, Cognitive[Title/Abstract])) OR (Cognitive Behavior Therapies[Title/Abstract])) OR (Therapies, Cognitive Behavior[Title/Abstract])) OR (Cognitive Psychotherapy[Title/Abstract])) OR (Cognitive Psychotherapies[Title/Abstract])) OR (Psychotherapies, Cognitive[Title/Abstract])) OR (Psychotherapy, Cognitive[Title/Abstract])) OR (Therapy, Cognitive[Title/Abstract])) OR (Cognitive Therapies[Title/Abstract])) OR (Therapies, Cognitive[Title/Abstract])) OR (Cognition Therapy[Title/Abstract])) OR (Therapy, Cognition[Title/Abstract])) OR (Cognition Therapies[Title/Abstract])) OR (Therapies, Cognition[Title/Abstract])) OR (Cognitive therapy[MeSH Terms])	87,204	3:23:14
		Behavior therapy	#8	(((((((((((Behaviour therapy[Title/Abstract]) OR (Behavior Therapies[Title/Abstract])) OR (Therapy, Conditioning[Title/Abstract])) OR (Conditioning Therapy[Title/Abstract])) OR (Conditioning Therapies[Title/Abstract])) OR (Therapy, Behavior[Title/Abstract])) OR (Behavior Treatment[Title/Abstract])) OR (Treatment, Behavior[Title/Abstract])) OR (Behavior Modification[Title/Abstract])) OR (Behavior Modifications[Title/Abstract])) OR (Modification, Behavior[Title/Abstract])) OR (Behaviour therapy[MeSH Terms])	98,627	3:22:36
		Cognitive behavioral therapy	#7	((((((((((((((((((((((((Cognitive behavioral therapy[Title/Abstract]) OR (Behavioral Therapies, Cognitive[Title/Abstract])) OR (Behavioral Therapy, Cognitive[Title/Abstract])) OR (Cognitive Behavioral Therapies[Title/Abstract])) OR (Therapies, Cognitive Behavioral[Title/Abstract])) OR (Therapy, Cognitive Behavioral[Title/Abstract])) OR (Therapy, Cognitive Behavior[Title/Abstract])) OR (Cognitive Behavior Therapy[Title/Abstract])) OR (Cognitive Therapy[Title/Abstract])) OR (Behavior Therapy, Cognitive[Title/Abstract])) OR (Behavior Therapies, Cognitive[Title/Abstract])) OR (Cognitive Behavior Therapies[Title/Abstract])) OR (Therapies, Cognitive Behavior[Title/Abstract])) OR (Cognitive Psychotherapy[Title/Abstract])) OR (Cognitive Psychotherapies[Title/Abstract])) OR (Psychotherapies, Cognitive[Title/Abstract])) OR (Psychotherapy, Cognitive[Title/Abstract])) OR (Therapy, Cognitive[Title/Abstract])) OR (Cognitive Therapies[Title/Abstract])) OR (Therapies, Cognitive[Title/Abstract])) OR (Cognition Therapy[Title/Abstract])) OR (Therapy, Cognition[Title/Abstract])) OR (Cognition Therapies[Title/Abstract])) OR (Therapies, Cognition[Title/Abstract])) OR (Cognitive behavioral therapy[MeSH Terms])	92,574	3:21:59
		Psychological intervention	#6	((((((((Psychological intervention[Title/Abstract]) OR (Intervention, Psychosocial[Title/Abstract])) OR (Interventions, Psychosocial[Title/Abstract])) OR (Psychosocial Interventions[Title/Abstract])) OR (Psychological Intervention[Title/Abstract])) OR (Intervention, Psychological[Title/Abstract])) OR (Interventions, Psychological[Title/Abstract])) OR (Psychological Interventions[Title/Abstract])) OR (Psychological intervention[MeSH Terms])	16,369	3:21:31
		Psychotherapy	#5	((Psychotherapy[Title/Abstract]) OR (Psychotherapies[Title/Abstract])) OR (Psychotherapy[MeSH Terms])	241,986	3:20:55
P	Stroke patients (excluding subarachnoid hemorrhage)		#4	Search (#1 or #2 or #3) (((((((((((Stroke[Title/Abstract]) OR (Cerebrovascular Accident[Title/Abstract])) OR (CVA[Title/Abstract])) OR (Cerebrovascular Apoplexy[Title/Abstract])) OR (Vascular Accident[Title/Abstract])) OR (Brain Vascular Accident[Title/Abstract])) OR (Cerebrovascular Stroke[Title/Abstract])) OR (Apoplexy[Title/Abstract])) OR (Cerebral Stroke[Title/Abstract])) OR (Acute Stroke[Title/Abstract])) OR (Acute Cerebrovascular Accident[Title/Abstract])) OR (Stroke[MeSH Terms])OR(((((((Brain Infarction[Title]) OR (Anterior Circulation Brain Infarction[Title])) OR (Venous Infarction[Title])) OR (Brain Venous Infarction[Title])) OR (Venous Brain Infarction[Title])) OR (Anterior Cerebral Circulation Infarction[Title])) OR (Posterior Circulation Brain Infarction[Title])) OR (Brain Infarction[MeSH Terms])OR((((((((((((Intracranial Hemorrhage[MeSH Terms]) OR (Intracranial Hemorrhage[Title/Abstract])) OR (Hemorrhages, Intracranial[Title/Abstract])) OR (Intracranial Hemorrhage[Title/Abstract])) OR (Hemorrhage, Intracranial[Title/Abstract])) OR (Posterior Fossa Hemorrhage[Title/Abstract])) OR (Hemorrhage, Posterior Fossa[Title/Abstract])) OR (Hemorrhages, Posterior Fossa[Title/Abstract])) OR (Posterior Fossa Hemorrhages[Title/Abstract])) OR (Brain Hemorrhage[Title/Abstract])) OR (Brain Hemorrhages[Title/Abstract])) OR (Hemorrhage, Brain[Title/Abstract])) OR (Hemorrhages, Brain[Title/Abstract])	464,155	3:20:05
		Intracranial hemorrhage	#3	((((((((((((Intracranial Hemorrhage[MeSH Terms]) OR (Intracranial Hemorrhage[Title/Abstract])) OR (Hemorrhages, Intracranial[Title/Abstract])) OR (Intracranial Hemorrhage[Title/Abstract])) OR (Hemorrhage, Intracranial[Title/Abstract])) OR (Posterior Fossa Hemorrhage[Title/Abstract])) OR (Hemorrhage, Posterior Fossa[Title/Abstract])) OR (Hemorrhages, Posterior Fossa[Title/Abstract])) OR (Posterior Fossa Hemorrhages[Title/Abstract])) OR (Brain Hemorrhage[Title/Abstract])) OR (Brain Hemorrhages[Title/Abstract])) OR (Hemorrhage, Brain[Title/Abstract])) OR (Hemorrhages, Brain[Title/Abstract])	94,510	0:00:00
		Brain infarction	#2	(((((((Brain Infarction[Title/Abstract]) OR (Anterior Circulation Brain Infarction[Title/Abstract])) OR (Venous Infarction[Title/Abstract)) OR (Brain Venous Infarction[Title/Abstract)) OR (Venous Brain Infarction[Title/Abstract])) OR (Anterior Cerebral Circulation Infarction[Title/Abstract])) OR (Posterior Circulation Brain Infarction[Title/Abstract])) OR (Brain Infarction[MeSH Terms])	46,490	3:18:21
		Stroke	#1	(((((((((((Stroke[Title/Abstract]) OR (Cerebrovascular Accident[Title/Abstract])) OR (CVA[Title/Abstract])) OR (Cerebrovascular Apoplexy[Title/Abstract])) OR (Vascular Accident[Title/Abstract])) OR (Brain Vascular Accident[Title/Abstract])) OR (Cerebrovascular Stroke[Title/Abstract])) OR (Apoplexy[Title/Abstract])) OR (Cerebral Stroke[Title/Abstract])) OR (Acute Stroke[Title/Abstract])) OR (Acute Cerebrovascular Accident[Title/Abstract])) OR (Stroke[MeSH Terms])	391,923	3:17:21

This scoping review included studies that focused on patients diagnosed with a stroke, combined upper limb interventions and psychological interventions, assessed upper limb motor dysfunction, were designed as RCTs, and were written in English.

Studies of patients diagnosed with subarachnoid hemorrhage or patients with upper limb motor dysfunction due to diseases other than stroke, as well as pharmacological studies, were excluded. The search for databases on psychological interventions was based on the results of a previous study by Knapp et al. [31].

Upper extremity functional interventions aimed at improving upper extremity functional recovery or reducing functional impairment (or both) were based on a previous study by Pollock et al. [32]. Psychotherapy was defined as lifestyle change support, coping support, social support, distress management (e.g., psychological interventions to address work or family stress, anxiety, or depression), relaxation therapy, or a combination of the aforementioned psychological interventions, based on a previous study by Albus et al. [33].

Study Selection

The first author (YI) searched the database. The initial screening aimed to narrow the number of articles by removing from the list all studies that were irrelevant or inappropriate. Duplicate articles were removed, and the remaining were reviewed by two reviewers (YI and ST) to identify the eligibility of titles and abstracts. Two authors (YI and ST) assessed the full text of all relevant articles. Disagreements during the article screening and selection screening phases were resolved by discussion, and a third person (TI) made the decisions. The interrater reliability agreement calculated by Cohen’s kappa is 0.63, which is considered high enough for interrater reliability.

Data Extraction Process

MS Excel (Microsoft Corporation, Redmond, Washington, United States) was used to prepare a sheet to extract data on participants, intervention methods, outcomes, and results.

Risk of Bias in Individual Studies

Two authors (YI and ST) assessed the risk of bias in the included studies using the PEDro scale [34]. The PEDro scale comprises 11 items (eligibility criteria, random allocation, concealed allocation, baseline comparability, blind participants, blind therapists, blind assessors, adequate follow-up, intention-to-treat analysis, between-group comparisons, and point estimates and variability) based on the Delphi list [35]. Each item is scored as either “yes” or “no.” Except for the “external validity” item, one point is added for each internal validity criterion met, and a maximum of 10 points can be scored using this scale. The PEDro score demonstrates moderate interrater reliability, with an intraclass correlation coefficient of 0.68 (95% confidence interval (CI): 0.57-0.76) for clinical trials [36].

Results 

**Figure 1:**
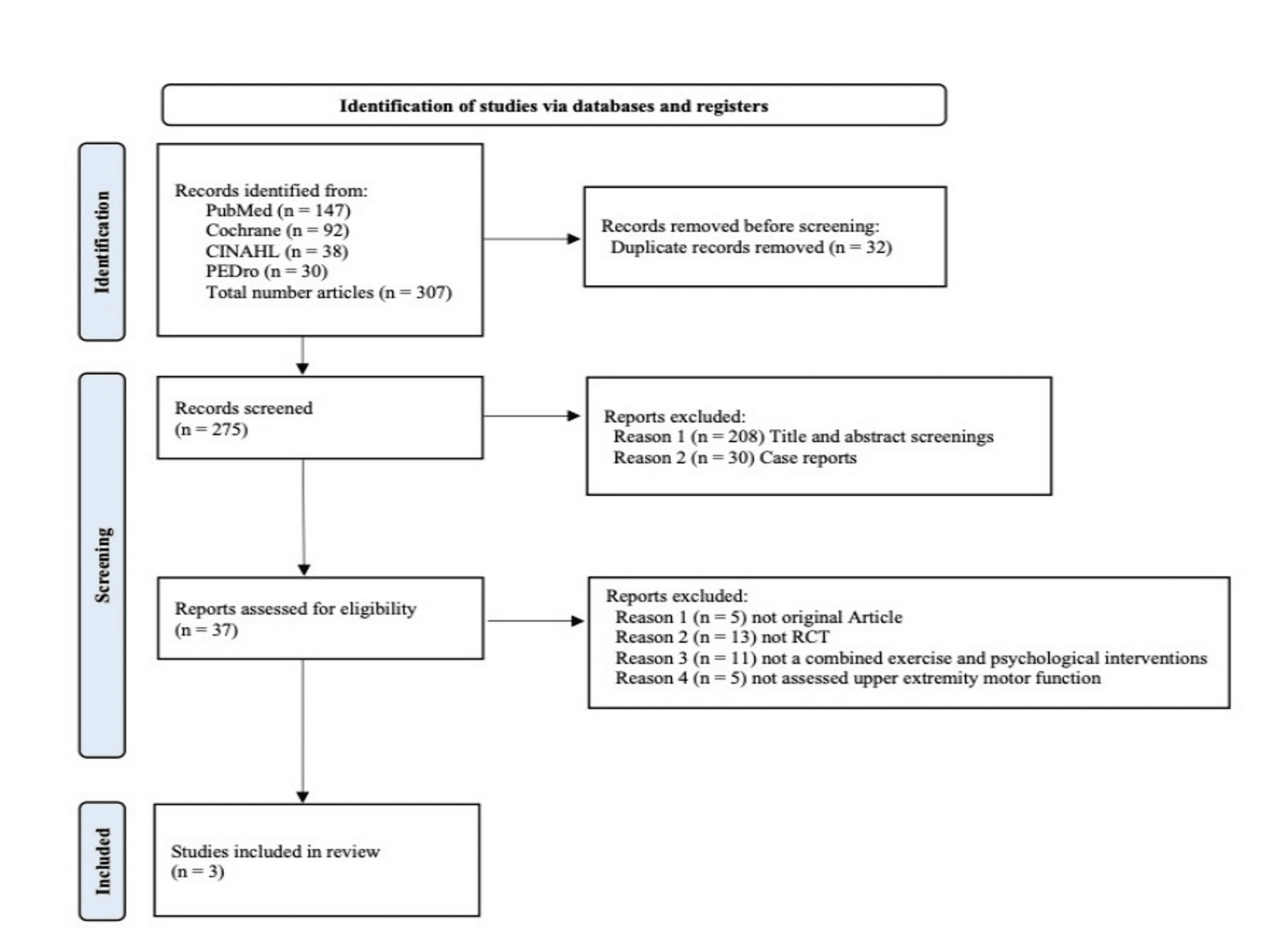
Preferred Reporting Items for Systematic Reviews and Meta-Analyses (PRISMA) flow diagram. PRISMA flow diagram for a scoping review exploring psychological interventions to improve upper limb motor dysfunction post-stroke

After sorting out duplicate papers, 307 studies remained. Among them, 270 studies did not meet the selection criteria after reviewing the title and abstract of the articles. The remaining 37 studies were reviewed in detail in full text, and 34 studies did not meet the selection criteria. Finally, three RCTs met the selection criteria. This full process is outlined in the PRISMA flowsheet (Figure 1).

Characteristics of the Included Studies

The final three RCTs included a total of 128 participants (Table 2). The psychological interventions examined in these RCTs comprised "COSTAR" in one study [37] and "CO-OP” in two studies [38,39]. The sample sizes of the included RCTs ranged from 35 to 49 participants, with the mean age of participants ranging from 54 to 62 years. Among the 128 participants, 68 received psychological interventions in conjunction with conventional upper limb function interventions, while 60 participants in the comparison group received only the upper limb interventions. Of the participants, 60.2% (n = 77) were male and 39.8% (n = 51) were female. Regarding the underlying conditions, 11.7% (n = 12) had a cerebral hemorrhage and 88.3% (n = 116) had an ischemic stroke. One RCT [37] did not report information on the paralyzed side of participants, while two RCTs [38,39] indicated that 63.1% (n = 53) and 36.9% (n = 31) of participants had right- and left-sided paralysis, respectively. Among the three RCTs, one study was conducted during the subacute to recovery phase (mean: <4 months post-stroke) [39], and two studies were conducted during the chronic phase (mean: >6 months post-stroke) [37,38]. Participants across these studies demonstrated moderate upper extremity motor dysfunction, although the specific evaluation measures varied among the studies. Reported dropout rates ranged from one to 27 participants, totaling 31 dropouts across the studies. One RCT [37] documented a 50% dropout rate in the combined intervention group (psychological and upper limb interventions) and a 25% dropout rate in the upper limb intervention group. Another RCT [38] reported no dropouts in the combined intervention group and a 4% dropout rate in the upper limb intervention group. The third RCT [39] reported dropout rates of 32% and 43% in the combined intervention group and the upper limb intervention group, respectively. Dropouts were attributed to the refusal of psychological interventions or the inability to complete evaluations after initiating the interventions.

**Table 2:** Characteristics of the participants in the target study TST: task-specific training; COSTAR: cognitive-oriented strategy training augmented rehabilitation; CO-OP: cognitive orientation to daily occupational performance; TUET: task-specific upper extremity training; UC: usual care, from “Combined Cognitive-Strategy and Task-Specific Training Improve Transfer to Untrained Activities in Subacute Stroke"; SIS: stroke impact scale; BBT: box-and-block test; WMFT-F: Wolf motor function test-functional score; ARAT: action research arm test

Study (author, journal, year)	Intervention	Size (n)	Age (years old)	Sex (M/F, n)	Stroke type (n)	Lesion side (R/L, n)	Time since stroke onset	Upper function at baseline
Wolf et al., Disabil Rehabil, 2021 [37]	TST＋COSTAR; TST	TST＋COSTAR (n = 24); TST (n = 20)	TST＋COSTAR 61.6 ± 10.2 years old; TST 58.8 ± 11.4 years old	TST＋COSTAR 12/12 (50%); TST 9/11 (45%)	TST＋COSTAR ischemia (n = 24); TST ischemia (n = 20)	Not reported	TST＋COSTAR 82.5 ± 91weeks; TST 61.9 ± 78.3 weeks	TST＋COSTAR SIS hand function 40.2 ± 21.7; TST SIS hand function 31.4 ± 24.4
Song et al., Restor Neurol Neurosci, 2019 [38]	CO-OP; TUET	CO-OP (n = 25); TUET (n = 24)	CO-OP 62.2 ± 14.7 years old; TUET 61.4 ± 14.8 years old	CO-OP 16/9 (64%); TUET 18/6 (75%)	CO-OP ischemia (n = 17) hemorrhage (n= 8); TUET ischemia (n = 17) hemorrhage (n = 7)	CO-OP 19/6; TUET 20/4	CO-OP 13.6 ± 5.8 months; TUET 14.6 ± 7.5 months	CO-OP BBT : 57.0 ± 8.8 WMFT-F : 22.3 ± 12.7; TUET BBT : 57.3 ± 9.6 WMFT-F : 31.1 ± 18.8
Wolf et al., AJOT, 2016 [39]	CO-OP; UC	CO-OP (n = 19); UC (n = 16)	CO-OP 57.5 ± 14.0 years old; UC 54.4 ± 14.0 years old	CO-OP 13/6 (68%); UC 9/7 (56%)	CO-OP ischemia (n = 19); UC ischemia (n = 16)	CO-OP 8/11; UC 3/13	CO-OP 40.1 ± 20.4 days; UC 46.5 ± 21.3 days	CO-OP SIS hand function 32.8 ± 27.0; UC SIS hand function 38.3 ± 26.7

Intervention Protocols and Settings

Psychological interventions were conducted once or twice per week over 12 sessions in the three included RCTs, with each session lasting between 30 and 45 minutes (Table 3). The total number of sessions per participant ranged from 12 to 20. The CO-OP intervention employed a Goal-Plan-Do-Check strategy to facilitate goal attainment as set by the participants. During this process, participants collaborated with therapists to define treatment goals. Participants subsequently worked on mastering each goal-oriented activity using the Goal-Plan-Do-Check problem-solving approach during the intervention sessions. Therapists facilitated problem-solving, adapted tasks to address performance challenges, and encouraged participants to engage in self-discovery.

**Table 3:** Interventions in the included studies TST: task-specific training; COSTAR: cognitive-oriented strategy training augmented rehabilitation; CO-OP: cognitive orientation to daily occupational performance; TUET: task-specific upper extremity training; UC: usual care, from “Combined Cognitive-Strategy and Task-Specific Training Improve Transfer to Untrained Activities in Subacute Stroke

Study (author, journal, year)	Exercise	Psychological intervention	Positions that provide psychological intervention	Intervention frequency	Intervention duration per session
Wolf et al., Disabil Rehabil, 2021 [37]	TST	COSTAR	Occupational therapist	TST＋COSTAR 1-2 times a week for 12 sessions; TST 1-2 times a week for 12 sessions	45 minutes
Song et al., Restor Neurol Neurosci, 2019 [38]	TUET	CO-OP	Occupational therapist; psychologist	CO-OP 5 times a week for 4 weeks; TUET 5 times a week for 4 weeks	30 minutes
Wolf et al., AJOT, 2016 [39]	UC	CO-OP	Occupational therapist	CO-OP 5 times a week for 4 weeks; UC 5 times a week for 4 weeks	30 minutes

The COSTAR intervention integrated the cognitive strategies of the CO-OP approach with task-specific training (TST) [37]. All psychological intervention sessions were conducted by either an occupational therapist or a psychologist. Control groups across the studies were provided with interventions based solely on TST-focused upper extremity interventions, which included [37] TST, [38] task-specific upper extremity training, and [39] usual care as part of the COSTAR protocol for improving transfer to untrained activities in subacute stroke.

Outcome Measures and Effects

The outcome measures of upper extremity motor dysfunction used in each of the three RCTs were different (Table 4). Wolf et al. [37,39] used the Stroke Impact Scale Hand Function (SIS hand function). Song et al. [38] utilized the box-and-block test (BBT) and the Wolf motor function test-functional score (WMFT-F). The SIS hand function measures the motor dysfunction degree of the paralyzed upper extremity in patients with post-stroke syndrome [40,41]. The BBT measures gross manual dexterity [42,43]. WMFT-F measures the degree of upper extremity motor dysfunction through functional tasks [44]. SIS hand function has been evaluated in two RCTs, but upper extremity motor dysfunction assessment had no commonalities in the three included RCTs. One RCT revealed that upper extremity combined with psychological intervention (CO-OP) significantly enhanced upper extremity motor dysfunction post-stroke compared to upper extremity intervention alone [38]. The second RCT revealed that upper extremity intervention combined with psychological intervention (CO-OP) was more effective than upper extremity intervention alone with moderate effect size (Period 1: d = 0.5; Period 2: d = 0.6) [39]. The third RCT revealed that upper extremity intervention alone was more effective than upper extremity combined with psychological interventions (COSTAR) (small effect size (d = 0.4)) [37]. The authors of all three RCTs [35-37] mentioned the study limitations in terms of sample size, differences in the time since stroke onset, and lack of blinding of outcome measures.

**Table 4:** Comparison of the results of the included studies TST: task-specific training; COSTAR: cognitive-oriented strategy training augmented rehabilitation; CO-OP: cognitive orientation to daily occupational performance; TUET: task-specific upper extremity training; UC: usual care, from “Combined Cognitive-Strategy and Task-Specific Training Improve Transfer to Untrained Activities in Subacute Stroke"; SIS: stroke impact scale; BBT: box-and-block test; WMFT-F: Wolf motor function test-functional score; d: Cohen’s d; effect size: 0.2 = small effect, 0.5 = medium effect, and 0.8 = large effect

Study (author, journal, year)	Data collection points	Outcome measures	Effect size	Results
Wolf et al., Disabil Rehabil, 2021 [37]	Period 1: the timing of discharge; Period 2: 9 months after discharge	SIS hand function	Period 1: d = 0.2; Period 2: d = 0.4	Mean scores for SIS hand function showed a small effect (d = 0.4) for the TST group compared to the TST + COSTAR group
Song et al., Restor Neurol Neurosci, 2019 [38]	After 4 weeks	BBT; WMFT-F	Not reported	The mean scores of WMFT-F and BBS were significantly higher in the CO-OP group than in the TUET group
Wolf et al., AJOT, 2016 [39]	Period 1: the timing of discharge; Period 2: 9 months after discharge	SIS hand function	Period 1: d = 0.5; Period 2: d = 0.6	Mean scores for SIS Hand Function showed a moderate effect (Period 1: d = 0.5, Period 2: d = 0.6) compared to usual occupational therapy

Assessment of the Risk of Bias

Of the three RCTs, one [37] scored 5 on the PEDro scale, and the remaining RCTs [38,39] scored 4 on the PEDro scale, both with fair risk of bias ratings (Table 5). All three RCTs correctly performed inclusion criteria, treatment and control group allocation, and statistical comparisons between groups, but points were lost for blinding of subjects and therapists.

**Table 5:** PEDro score results included studies PEDro: the Physiotherapy Evidence Database; C1: eligibility criteria were specified; C2: subjects were randomly allocated an order in which treatments were received; C3: allocation was concealed; C4: the groups were similar at baseline regarding the most important prognostic indicators; C5: all subjects were blinded; C6: there was blinding of all therapists who administered the therapy; C7: there was blinding of all assessors who measured at least one key outcome; C8: measures of at least one key outcome were obtained from more than 85% of the subjects initially allocated to groups; C9: all subjects for whom outcome measures were available received the treatment or control condition as allocated or, where this was not the case, data for at least one key outcome was analyzed by “intention to treat.” C10: the results of between-group statistical comparisons are reported for at least one key outcome; C11: the study provides both point measures and measures of variability for at least one key outcome; Score and quality rating 9–10: excellent methodological quality; 6–8: good methodological quality; 4–5: fair methodological quality; 0–3: poor methodological quality

Study	C1	C2	C3	C4	C5	C6	C7	C8	C9	C10	C11	PEDro score
Wolf et al., Disabil Rehabil, 2021 [37]	Yes	No	No	Yes	No	No	Yes	No	Yes	Yes	Yes	5 / 10
Song et al., Restor Neurol Neurosci, 2019 [38]	Yes	No	No	No	No	No	Yes	No	Yes	Yes	Yes	4 / 10
Wolf et al., AJOT, 2016 [39]	Yes	No	No	No	No	No	Yes	No	Yes	Yes	Yes	4 / 10

Discussion 

The present study comprehensively investigated the usefulness of combined upper extremity and psychological interventions in improving upper extremity motor dysfunction in patients with post-stroke syndrome. The psychological interventions in the three RCTs [37-39] that matched the search results were CO-OP and COSTAR, methods that increase the acquisition of problem-solving skills through cognitive strategies. The three RCTs [37-39] revealed different results with no clear conclusions regarding the usefulness of combined psychological and upper limb interventions in improving post-stroke upper limb motor dysfunction. Conversely, two RCTs [38,39] revealed that the combination of psychological, CO-OP, and upper extremity interventions was effective in improving post-stroke upper extremity motor dysfunction. The risk of bias assessed by the PEDro scale was fair quality in all three articles.

Previous studies revealed that psychological interventions improved upper extremity motor dysfunction in patients [45]. However, to the best of our knowledge, no previous scoping reviews, systematic reviews, or clinical practice guidelines have investigated the usefulness of a combination of upper extremity and psychological interventions for improving post-stroke upper extremity motor dysfunction. Therefore, the types of psychological interventions used in conjunction with upper extremity interventions to improve post-stroke upper extremity motor dysfunction and their usefulness are unclear. This scoping review is the first study to summarize the use of upper extremity interventions in combination with psychological interventions for post-stroke upper extremity motor dysfunction.

The psychological interventions in the three RCTs [37-39] included CO-OP and COSTAR, both of which use cognitive strategies to facilitate problem-solving to determine cognitive strategies that improve performance [46]. The main goals of CO-OP are to develop new skills, learn cognitive strategies, apply learned skills to real-world situations, and transfer learned skills and strategies to other activities [38,39]. Systematic CO-OP reviews revealed positive effects on the implementation of activities in children [47]. COSTAR combines the cognitive strategies of CO-OP with TST [37]. COSTAR is best described as an evidence-based, patient-centered, and TST-based rehabilitation protocol augmented with metacognitive strategy use. CO-OP and COSTAR use guided discovery rather than explicit instruction by the therapist to identify these strategies through an iterative process that helps patients analyze their performance and discover domain-specific strategies that resolve performance problems. We followed Pollock et al.’s definition of psychological interventions [31] and defined CO-OP and COSTAR as psychological interventions because they provide “lifestyle change support” and “coping support.”

The combination of psychological intervention CO-OP and upper extremity intervention was more effective than upper extremity intervention alone in enhancing post-stroke upper extremity motor dysfunction [38,39]. However, upper extremity intervention alone was more effective than the combination of psychological intervention COSTAR and upper extremity intervention in improving post-stroke upper extremity motor dysfunction [37]. The authors [37] indicate that the combined psychological (COSTAR) and upper extremity intervention group was less effective than the upper extremity intervention alone because of the higher number of patients with depression at baseline in the combined psychological (COSTAR) and upper extremity intervention group (n = 13) than in the upper extremity intervention alone (n = 5), which may act as a confounding variable for improvement in upper motor dysfunction. Additionally, CO-OP and COSTAR focus on participant activity and goal setting and are unnecessarily specific to improving upper extremity motor dysfunction [37-39]. Therefore, they are not necessarily effective in improving upper limb motor dysfunction when compared to programs, such as TST alone, that focus specifically on improving upper limb motor dysfunction. Moreover, TST is a program dedicated to improving upper limb motor dysfunction through tasks; thus, one RCT [37] revealed that interventions targeting only the upper limb were effective in improving upper limb motor dysfunction regardless of psychological interventions.

Limitations and directions for future research

This scoping review had several limitations. First, in this study, psychological interventions aimed at improving upper extremity motor dysfunction in patients with post-stroke syndrome included interventions that worked on cognitive aspects, but not interventions that directly involved mental aspects (e.g., emotion, motivation). Previous studies [48,49] revealed that increasing patient motivation is important for improving motor dysfunction. However, we were unable to investigate interventions other than cognitive interventions since this review only included studies that addressed cognitive aspects. Second, only three RCTs were applicable and no quantitative evaluation was conducted; thus, no clear conclusions were drawn regarding the usefulness of combining upper extremity and psychological interventions. In the future, a meta-analysis can evaluate the quality of evidence on the usefulness of upper extremity and psychological interventions when similar RCTs are accumulated. Third, CO-OP and COSTAR primarily emphasize participants’ activities and goal setting, even when combining psychological with physical interventions through cognitive strategies, and are not necessarily specialized in improving upper limb motor function impairment. Consequently, the effective interaction between cognitive psychological interventions and physical functional improvement may not be fully used. Therefore, the results of this review revealed no clear conclusion regarding the usefulness of combining upper extremity and psychological interventions in improving post-stroke upper limb motor function impairment.

## Conclusions

The included psychological interventions in this scoping review were CO-OP in two articles and COSTAR in one article. Additionally, two RCTs indicated that the combination of CO-OP and upper extremity intervention was more effective than upper extremity intervention alone. CO-OP and COSTAR were cognitive strategy-based interventions only, primarily focusing on participant activity and goal setting and not necessarily on improving upper extremity motor dysfunction. The results of this study provide no definitive conclusions regarding the usefulness of combining upper extremity and psychological interventions in improving post-stroke upper extremity motor dysfunction, but this is the first scoping review to map the utility of upper extremity interventions combined with psychological interventions.

## References

[1] Bonita R, Ford MA, Stewart AW (1988). Predicting survival after stroke: a three-year follow-up. Stroke.

[2] Broeks JG, Lankhorst GJ, Rumping K, Prevo AJ (1999). The long-term outcome of arm function after stroke: results of a follow-up study. Disabil Rehabil.

[3] Lai SM, Studenski S, Duncan PW (2002). Persisting consequences of stroke measured by the stroke impact scale. Stroke.

[4] Lewthwaite R, Winstein CJ, Lane CJ (2018). Accelerating stroke recovery: body structures and functions, activities, participation, and quality of life outcomes from a large rehabilitation trial. Neurorehabil Neural Repair.

[5] Campbell BC, Khatri P (2020). Stroke. Lancet.

[6] Franceschini M, La Porta F, Agosti M (2010). Is health-related-quality of life of stroke patients influenced by neurological impairments at one year after stroke?. Eur J Phys Rehabil Med.

[7] Sveen U, Bautz-Holter E, Sødring KM, Wyller TB, Laake K (1999). Association between impairments, self-care ability and social activities 1 year after stroke. Disabil Rehabil.

[8] Heller A, Wade DT, Wood VA, Sunderland A, Hewer RL, Ward E (1987). Arm function after stroke: measurement and recovery over the first three months. J Neurol Neurosurg Psychiatry.

[9] Nakayama H, Jørgensen HS, Raaschou HO (1994). Recovery of upper extremity function in stroke patients: the Copenhagen stroke study. Arch Phys Med Rehabil.

[10] Kleim JA, Jones TA (2008). Principles of experience-dependent neural plasticity: implications for rehabilitation after brain damage. J Speech Lang Hear Res.

[11] Daly JJ, Ruff RL (2007). Construction of efficacious gait and upper limb functional interventions based on brain plasticity evidence and model-based measures for stroke patients. ScientificWorldJournal.

[12] Platz T (2021). Clinical Pathways in Stroke Rehabilitation: Evidence-based Clinical Practice Recommendations.

[13] Kneebone II, Dunmore E (2000). Psychological management of post-stroke depression. Br J Clin Psychol.

[14] Onyike CU (2006). Cerebrovascular disease and dementia. Int Rev Psychiatry.

[15] Cumming TB, Marshall RS, Lazar RM (2013). Stroke, cognitive deficits, and rehabilitation: still an incomplete picture. Int J Stroke.

[16] Sinyor D, Amato P, Kaloupek DG, Becker R, Goldenberg M, Coopersmith H (1986). Post-stroke depression: relationships to functional impairment, coping strategies, and rehabilitation outcome. Stroke.

[17] Hama S, Yoshimura K, Yanagawa A (2020). Relationships between motor and cognitive functions and subsequent post-stroke mood disorders revealed by machine learning analysis. Sci Rep.

[18] White JH, Attia J, Sturm J, Carter G, Magin P (2014). Predictors of depression and anxiety in community dwelling stroke survivors: a cohort study. Disabil Rehabil.

[19] Gurr B, Muelenz C (2011). A follow-up study of psychological problems after stroke. Top Stroke Rehabil.

[20] Dancause N (2006). Vicarious function of remote cortex following stroke: recent evidence from human and animal studies. Neuroscientist.

[21] Hosp JA, Pekanovic A, Rioult-Pedotti MS, Luft AR (2011). Dopaminergic projections from midbrain to primary motor cortex mediate motor skill learning. J Neurosci.

[22] Miller EL, Murray L, Richards L, Zorowitz RD, Bakas T, Clark P, Billinger SA (2010). Comprehensive overview of nursing and interdisciplinary rehabilitation care of the stroke patient: a scientific statement from the American Heart Association. Stroke.

[23] (2018). VA/DOD Clinical Practice Guideline for the Management of Posttraumatic Stress Disorder and Acute Stress Disorder: Clinician Summary. Focus (Am Psychiatr Publ).

[24] Kampling H, Reese C, Küst J, Mittag O (2020). Systematic development of practice guidelines for psychological interventions in stroke rehabilitation. Disabil Rehabil.

[25] Merriman NA, Gillan D, Pender N (2021). The StrokeCog study: development and description of a cognition-focused psychological intervention to address cognitive impairment following stroke. Psychol Health.

[26] Gunnes M, Langhammer B, Aamot IL (2019). Adherence to a long-term physical activity and exercise program after stroke applied in a randomized controlled trial. Phys Ther.

[27] Therrien AS, Wolpert DM, Bastian AJ (2016). Effective reinforcement learning following cerebellar damage requires a balance between exploration and motor noise. Brain.

[28] Pohl PS, Filion DL, Kim SH (2003). Effects of practice and unpredictable distractors on planning and executing aiming after stroke. Neurorehabil Neural Repair.

[29] Juckett LA, Wengerd LR, Faieta J, Griffin CE (2020). Evidence-based practice implementation in stroke rehabilitation: A scoping review of barriers and facilitators. Am J Occup Ther.

[30] Damschroder LJ, Aron DC, Keith RE, Kirsh SR, Alexander JA, Lowery JC (2009). Fostering implementation of health services research findings into practice: a consolidated framework for advancing implementation science. Implement Sci.

[31] Knapp P, Campbell Burton CA, Holmes J (2017). Interventions for treating anxiety after stroke. Cochrane Database Syst Rev.

[32] Pollock A, Farmer SE, Brady MC (2014). Interventions for improving upper limb function after stroke. J Paramed Pract.

[33] Albus C, Herrmann-Lingen C, Jensen K (2019). Additional effects of psychological interventions on subjective and objective outcomes compared with exercise-based cardiac rehabilitation alone in patients with cardiovascular disease: a systematic review and meta-analysis. Eur J Prev Cardiol.

[34] De Morton NA (2009). The PEDro scale is a valid measure of the methodological quality of clinical trials: a demographic study. Aust J Physiother.

[35] Atkins D, Eccles M, Flottorp S (2004). Systems for grading the quality of evidence and the strength of recommendations I: critical appraisal of existing approaches The GRADE Working Group. BMC Health Serv Res.

[36] Maher CG, Sherrington C, Herbert RD (2003). Reliability of the PEDro scale for rating quality of randomized controlled trials. Phys Ther.

[37] Wolf TJ, Doherty M, Boone A, Rios J, Polatajko H, Baum C, McEwen S (2021). Cognitive oriented strategy training augmented rehabilitation (COSTAR) for ischemic stroke: a pilot exploratory randomized controlled study. Disabil Rehabil.

[38] Song CS, Lee ON, Woo HS (2019). Cognitive strategy on upper extremity function for stroke: a randomized controlled trials. Restor Neurol Neurosci.

[39] Wolf TJ, Polatajko H, Baum C, Rios J, Cirone D, Doherty M, McEwen S (2016). Combined cognitive-strategy and task-specific training affects cognition and upper-extremity function in subacute stroke: an exploratory randomized controlled trial. Am J Occup Ther.

[40] Duncan PW, Wallace D, Lai SM, Johnson D, Embretson S, Laster LJ (1999). The stroke impact scale version 2.0: evaluation of reliability, validity, and sensitivity to change. Stroke.

[41] Duncan PW, Wallace D, Studenski S, Lai SM, Johnson D (2001). Conceptualization of a new stroke-specific outcome measure: the stroke impact scale. Top Stroke Rehabil.

[42] Mathiowetz V, Volland G, Kashman N, Weber K (1985). Adult norms for the box and block test of manual dexterity. Am J Occup Ther.

[43] Desrosiers J, Bravo G, Hébert R, Dutil E, Mercier L (1994). Validation of the box and block test as a measure of dexterity of elderly people: reliability, validity, and norms studies. Arch Phys Med Rehabil.

[44] Wolf SL, Catlin PA, Ellis M, Archer AL, Morgan B, Piacentino A (2001). Assessing Wolf motor function test as outcome measure for research in patients after stroke. Stroke.

[45] Widmer M, Held JP, Wittmann F, Lambercy O, Lutz K, Luft AR (2017). Does motivation matter in upper-limb rehabilitation after stroke? ArmeoSenso-Reward: study protocol for a randomized controlled trial. Trials.

[46] Scammell EM, Bates SV, Houldin A, Polatajko HJ (2016). The cognitive orientation to daily occupational performance (CO-OP): a scoping review. Can J Occup Ther.

[47] Madieu E, Gagné-Trudel S, Therriault PY, Cantin N (2023). Effectiveness of CO-OP approach for children with neurodevelopmental disorders: a systematic review. Arch Rehabil Res Clin Transl.

[48] Hatem SM, Saussez G, Della Faille M, Prist V, Zhang X, Dispa D, Bleyenheuft Y (2016). Rehabilitation of motor function after stroke: a multiple systematic review focused on techniques to stimulate upper extremity recovery. Front Hum Neurosci.

[49] Molina-Luna K, Hertler B, Buitrago MM, Luft AR (2008). Motor learning transiently changes cortical somatotopy. Neuroimage.

